# Experimental Design Optimization of Acrylate—Tannin Photocurable Resins for 3D Printing of Bio-Based Porous Carbon Architectures

**DOI:** 10.3390/molecules27072091

**Published:** 2022-03-24

**Authors:** Pauline Blyweert, Vincent Nicolas, Vanessa Fierro, Alain Celzard

**Affiliations:** Institut Jean Lamour, Université de Lorraine, UMR 7198, 27 Rue Philippe Seguin, CEDEX 9, 88051 Epinal, France; pauline.blyweert@univ-lorraine.fr (P.B.); vincent.nicolas@univ-lorraine.fr (V.N.); vanessa.fierro@univ-lorraine.fr (V.F.)

**Keywords:** tannin, porous carbon, stereolithography, additive manufacturing, experimental design

## Abstract

In this work, porous carbons were prepared by 3D printing formulations based on acrylate–tannin resins. As the properties of these carbons are highly dependent on the composition of the precursor, it is essential to understand this effect to optimise them for a given application. Thus, experimental design was applied, for the first time, to carbon 3D printing. Using a rationalised number of experiments suggested by a Scheffé mixture design, the experimental responses (the carbon yield, compressive strength, and Young’s modulus) were modelled and predicted as a function of the relative proportions of the three main resin ingredients (HDDA, PETA, and CN154CG). The results revealed that formulations containing a low proportion of HDDA and moderate amounts of PETA and CN154CG gave the best properties. Thereby, the optimised carbon structures had a compressive strength of over 5.2 MPa and a Young’s modulus of about 215 MPa. The reliability of the model was successfully validated through optimisation tests, proving the value of experimental design in developing customisable tannin-based porous carbons manufactured by stereolithography.

## 1. Introduction

3D printing opens up new design possibilities for the fabrication of complex carbon materials with considerable application potential with specific customisation [1,2]. Obtaining a porous carbon material in 3D through a multi-step process, i.e., carbon precursor formulation, printing, and pyrolysis, is easily achievable with stereolithography (SLA), one of the most accurate 3D printing technologies [3,4]. Existing studies have allowed the realisation of highly porous carbon materials that have been primarily exploited for applications in electrochemistry and for which the carbon architecture and the carbonisation temperature have been the main parameters studied [5,6,7]. 

However, the direct relationships between the photocurable formulations, usually based on acrylates, and the properties of these carbons, especially the mechanical properties, have not been studied thus far, and only general trends have been observed (i.e., the increase of the properties with density [8] or the effect of anisotropy due to the printing technology [9]). To this end, it is essential to understand the effects of the various components of the resin formulation on the final properties of the carbons. In addition, in most cases, the printed resin has a low carbon yield upon pyrolysis and is of purely petrochemical origin [5,6,7,10,11,12]. Thus, to make 3D-printed porous carbons more sustainable and improve their yield, it is interesting to investigate partially bio-based resins, such as acrylate–tannin resins.

Condensed tannins are cheap and abundant phenolic bioresources that have been extensively used and studied to prepare a wide range of carbon materials (i.e., monoliths, gels, and nanoparticles, among others) [13,14,15]. They are not polymerisable under UV light [16] but have proven their potential as a carbon precursor filler in acrylate resins designed for SLA. Thus far, the effect of the tannin content on the mechanical properties of the derived carbons has been studied [17]. However, a description of the impact of the acrylate formulation on the properties of the final porous carbon is still lacking. Therefore, the main objective of the present work was to investigate the relationship between precursor formulation and mechanical properties and carbon yield. 

The final goal is to develop 3D porous tannin-based carbons that are easily manipulated without the risk of breakage, a property that many non-graphitic carbons lack. In order to achieve this objective, 3D-printed porous carbon architectures based on acrylate-tannin resin were prepared and analysed in a rational way using, for the first time with this kind of material, an experimental design and related statistical analysis. Design of Experiments (DOE) is an efficient methodology used to organise the most relevant experiments and obtain information on complex systems whose properties depend on the composition or process [18,19].

## 2. Experimental Section

### 2.1. Materials

The acrylated aromatic oligomer (CN154 CG), the acrylated aliphatic tetraacrylate (PETA; SR295), and the reactive diluent (HDDA; SR238) were kindly provided by Sartomer (Arkema Group, Verneuil en Halatte, France). The radical photoinitiator bis(2,4,6-trimethylbenzoyl)phenylphosphine oxide (BAPO) was supplied by Lambson (Arkema Group, Wetherby, England). Mimosa tannin extract (from Accacia Mearnsii), known as Fintan OP on the market, was kindly provided by SilvaChimica (St Michele Mondovi, Italy) and used as a bio-based carbon precursor. All products were used as received without further purification.

### 2.2. Preparation of 3D-Printed Carbon Architectures Based on Acrylate–Tannin Resins

**Figure 1:**
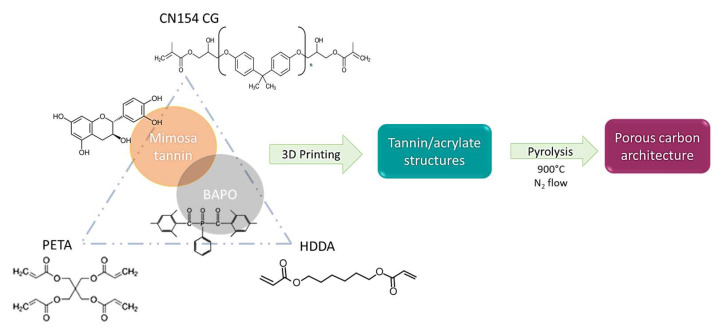
Scheme of the synthesis method of 3D-printed tannin-based carbon architectures.

The acrylate–tannin resin formulations were produced as follows (see Figure 1). A BAPO initiator was first dissolved in HDDA by vigorous stirring. Then, PETA and CN154 CG were added and homogenised in a heated ultrasonic bath (50 °C, 20 min). Finally, tannin was added and vigorously stirred for about 2 min to ensure good dispersion in the resin. 

Table 1 gives a representative example of a typical acrylate–tannin resin composition prepared in this work. Tannins are not polymerisable under UV light; therefore, only the acrylate components are involved in the free radical polymerisation that occurs during 3D printing [20].

**Figure 2:**
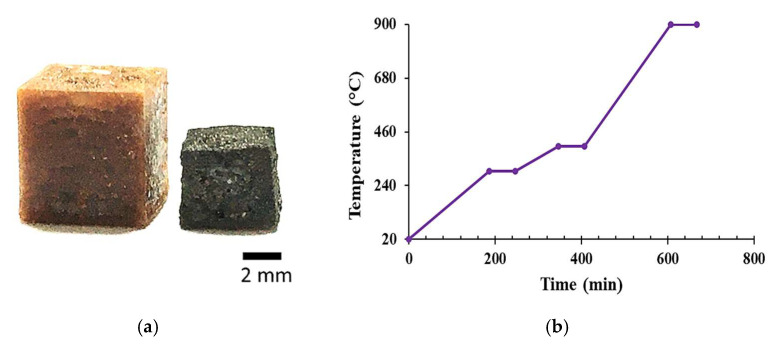
(**a**) Example of 3D-printed structures before (**left**) and after pyrolysis (**right**). (**b**) Heating ramp for the pyrolysis step.

The resultant resins were then processed in a DWS J28 (desktop SLA; 405 nm) high-resolution 3D printer to print four plain cubes (overall size 7 × 7 × 7 mm; Figure 2). The printed objects were post-cured in a UV oven (405 nm at 35 W) for 20 min at room temperature and were finally converted into highly disordered carbon materials [17] under pure nitrogen in a tubular furnace with a heating ramp of 1.5 °C·min^−1^ to 300 °C, with a 60 min step at 300 °C, a second ramp of 1 °C·min^−1^ to 400 °C (60 min step), followed by a third ramp of 2 °C min^−1^ to a final temperature of 900 °C, which was maintained for 1 h. 

We observed that the addition of two isotherms at 300 and 400 °C allowed for a better release of gases produced by the thermal degradation of the resin and considerably reduced cracking during carbonisation. Thus, this temperature program led to a better stabilisation of the structures during mass loss. Figure 2 shows an example of a 3D-printed structure before and after pyrolysis. The linear shrinkage during pyrolysis was similar for all formulations (about 22%), and the final size of the carbon structures was, on average, 5.2 × 5.2 × 5.2 mm.

### 2.3. Experimental Design

In order to understand the effects of the resin composition among a number of possible formulations, the concepts of experimental design and statistical analysis were applied [19]. This methodology allows rationalising and saves a great deal of time and material to obtain information on systems whose properties depend on the composition and processing conditions [18,21]. Therefore, the present study was conducted to document the ability of such a method to map the properties/formulation relationships and optimise a UV-curable resin for the 3D printing of porous carbons to improve their mechanical properties and yield.

UV-curable resins for 3D printing include an oligomer (CN154CG, whose fraction here will be referred to as *A*), a reactive monomer (PETA, referred to as *B*), a reactive diluent (HDDA, referred to as *C*), a photoinitiator, and other additives—in our case, a carbon precursor (condensed mimosa tannin). The most important parameter in the formulation of the acrylate–tannin resin for stereolithography is the viscosity of the resin. The operability limit is usually set at 3 Pa·s (at printing temperature, about 25 °C and at a shear of about 100 s^−1^) [22,23]. 

The acrylate fractions, at a fixed tannin content, used to prepare 3D-printed carbons are therefore influenced by the region of process operability [24]. For instance, too high a fraction of CN154 CG (viscosity of 1.55–2.50 Pa·s at 65 °C) will drastically increase the viscosity of the resin above 3 Pa·s so that correct printing will be impossible. Constraints on the acrylate contents were set to reduce the viscosity of the resin from a common formulation ratio: 0.4 oligomer/0.4 reactive monomer/0.2 reactive diluent [17,25].

The effect of too low a viscosity on the stability of the acrylate-tannin suspension was not considered here as the printed structure required a short printing time; however, some additional constraints and processing parameters, such as printing settings [26,27,28], could have entered the experimental design. The acrylates were chosen as variables, their total representing 74.7 wt.% of the final resin, while the tannin (25 wt.%) and photoinitiator (0.3 wt.%) contents were equal in all formulations. 

The carbon yield, compressive strength, and Young’s modulus were chosen as responses for obvious reasons: (i) yield is an absolutely essential parameter because most photocrosslinkable resins have an almost zero yield upon pyrolysis. It is therefore impossible to obtain carbonaceous materials directly by pyrolysing them if no additives (such as tannin) are used. Moreover, other properties, such as porosity and shrinkage are consequences of this. (ii) Mechanical properties are essential data for characterising materials, especially when it comes to the development of new materials that are expected to be not overly brittle in use. As these are so-called hard carbons because of their non-graphical nature, compression tests were naturally the most suitable.

In an experimental design, it is not the actual amount of each acrylate that matters, rather, its proportion to the other variable ingredients. Thus, the sum of the three acrylates compounds remains constant and equal to 1 in the mixture design (Table 2) but corresponds to 74.7 wt.% of the print resin. Once the aforementioned boundaries are defined, the experimental design problem can be investigated. The relationships between the proportions of the three ingredients used in the problem are shown in the set of Equation (1) where *A*, *B*, and *C* are the fractions of CN154CG, PETA, and HDDA, respectively, and *Y_i_* is the response: the carbon yield, compressive strength, or Young’s modulus.

(1) $$\{\begin{array}{c} Y_{i}=f\left(A,B,C\right)\\ A+B+C=1\;\\ 0\leq A\leq 0.4\\ 0\leq B\leq 0.4\\ 0.2\;\leq C\leq 1 \end{array}$$

**Figure 3:**
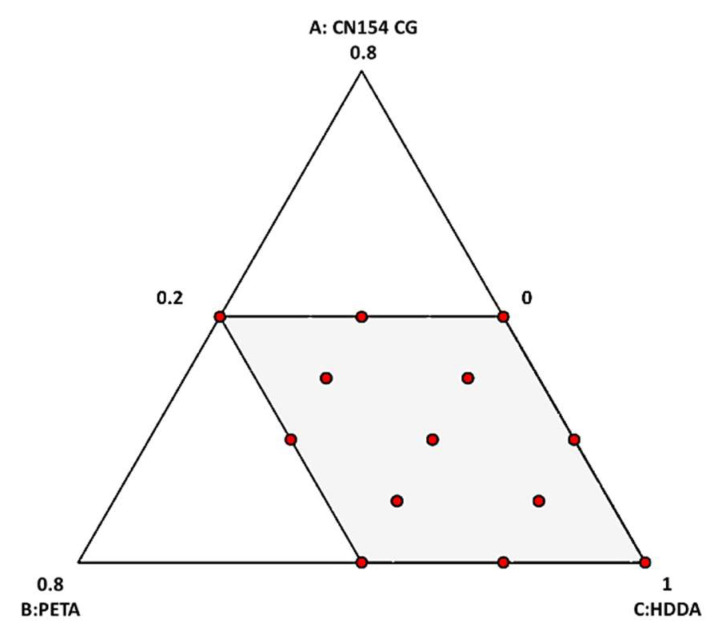
Distribution of experiment design points in the investigated range of parameters. A: CN154CG, B: PETA, and C: HDDA.

Therefore, the present study was performed entirely using the Design-Expert^®^ 13 software (Stat-Ease, Inc., Minneapolis, MN, USA). The set of experiments conducted in this study included eight factorial points, four middle boundary edges, and one central point according to a special cubic model; see Table 2 and Figure 3.

In the following paragraphs, carbon structures are named after the formulation number of the resin from which they are derived.

### 2.4. Characterisation of 3D-Printed Carbon Architectures

The samples were characterised in terms of their chemical composition, structure, carbon yield, bulk density, porosity, and mechanical properties. The elemental analysis (EA) of the carbon materials was performed in an Elementar Vario EL Cube analyser where the bulk contents of sulphur, nitrogen, hydrogen, and carbon were measured. The oxygen content was calculated by the difference.

Transmission Electron Microscopy (TEM) images were obtained using a JEM ARM 200F Cold FEG TEM/STEM operating at 200 kV and equipped with a spherical aberration (Cs) probe and image correctors (point resolution 0.12 nm in TEM mode and 0.078 nm in STEM mode). The samples were prepared by dispersing carbon powder in ethanol, after which a single drop of the resultant suspension was deposited on a carbon-coated copper grid (200 mesh).

The bulk density of the carbon architectures, *ρ_b_* (g·cm^−3^), defined as the mass of the materials divided by their total volume, was simply obtained by weighing the aforementioned samples of known dimensions and calculating the average. The skeletal density, *ρ_s_* (g·cm^−3^), was determined by helium pycnometry using an automatic Accupyc II 1340 (Micromeritics) apparatus by averaging 30 measurements for each carbon sample. Each sample was ground in a mortar to avoid neglecting a possible closed porosity fraction and then dried at 105 °C for several hours before measurement. From the experimental values of the bulk and skeletal densities, the total porosity *Φ* (dimensionless) was calculated according to Equation (2).

(2) $$\Phi =1-\frac{\rho_{b}}{\rho_{s}}$$

Mercury intrusion was performed using a Micromeretics AutoPore IV 9500 apparatus. The experiments were conducted in two steps: first in a low-pressure chamber in the pressure range of 0.001 to 0.24 MPa, and then in a high-pressure chamber in the pressure range of 0.245 to 414 MPa. The pore entrance diameter, D (m), was calculated by application of the Washburn Equation (3):

(3) $$D=-\frac{4\gamma \;\cos\varphi }{P}$$

where *γ* (480 mJ·m^−2^) is the surface tension of mercury, *φ* (140°) is the contact angle between mercury and most solid materials, and *P* (Pa) is the intrusion pressure.

X-ray tomography was performed with a RX Solution tomograph driven by X-Act software. The X-ray source voltage was set to 130 kV and the current to 30 µA. The measurements were recorded as 2D images arrays with isotropic voxel sizes of 4.9 µm. The reconstructed images were analysed using ImageJ (open source software).

The mechanical tests were conducted in triplicate at a constant compression rate (2 mm min^−1^) with an Instron 5944 universal testing machine equipped with a 2 kN load cell. The samples were tested in the printing direction (z-direction, i.e., orthogonal to the successive layers) and were previously bonded to PMMA plates with a very thin layer of epoxy. During the tests, the deformation (mm) and applied force (N) were continuously recorded and converted into strain (%) and stress (MPa) from the known dimensions of the samples. 

The Young’s modulus, defined as the slope of the initial linear part of the curve presenting the steepest slope, and the compressive strength at break were estimated from the stress–strain curves obtained. The typical stress–strain curve of brittle cellular materials, such as porous carbon monoliths, generally shows a linear elastic zone at very low strain, followed by a long, more or less horizontal, saw-toothed, and highly noisy plateau, corresponding to the successive collapse of different cell layers. When the cells have almost completely collapsed, a densification step can be observed in which the stress increases sharply with further strain [29].

## 3. Results and Discussion

### 3.1. Properties of 3D-Printed Carbons

The bulk chemical composition of the different 3D-printed carbons was similar for all samples with very low nitrogen (N) content, no sulphur (S), and a significant amount of oxygen (O) in the carbon structures. No significant differences were observed between the compositions of the samples, with C, H, N, S, and O contents of about 95, 1.3, 0.3, 0, and 3.5 wt.%, respectively, which is in agreement with the composition of other carbons based on mimosa tannin [13,30].

**Figure 4:**
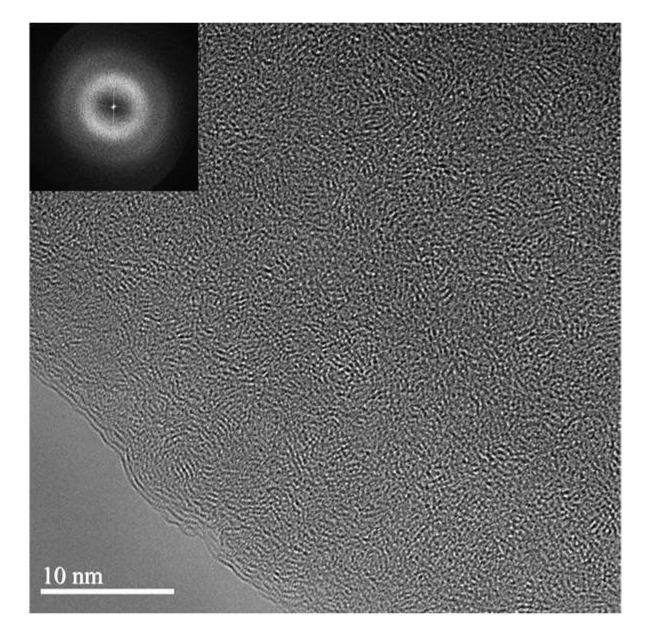
High-resolution TEM image of a 3D-printed carbon structure. The inset shows the corresponding Fast Fourier Transform (FFT) image.

TEM observations were performed to investigate the nanostructure of the carbon materials (Figure 4). As expected in the absence of a graphitisation catalyst [13], TEM revealed highly disordered structures for the different samples. The chemical and structural properties did not show significant differences between the 3D-printed carbons, hence, indicating the importance of the macro scale characterisation.

**Figure A1:**
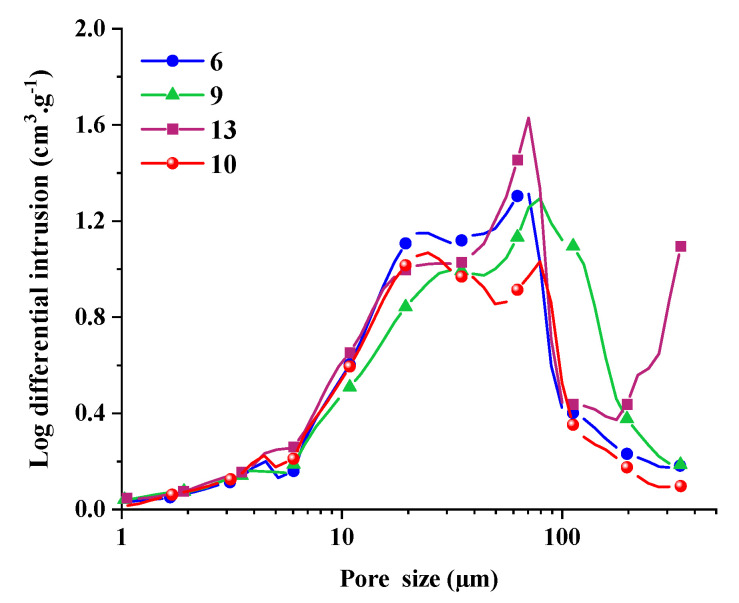
Pore size distribution calculated using Equation (3).

**Figure A2:**
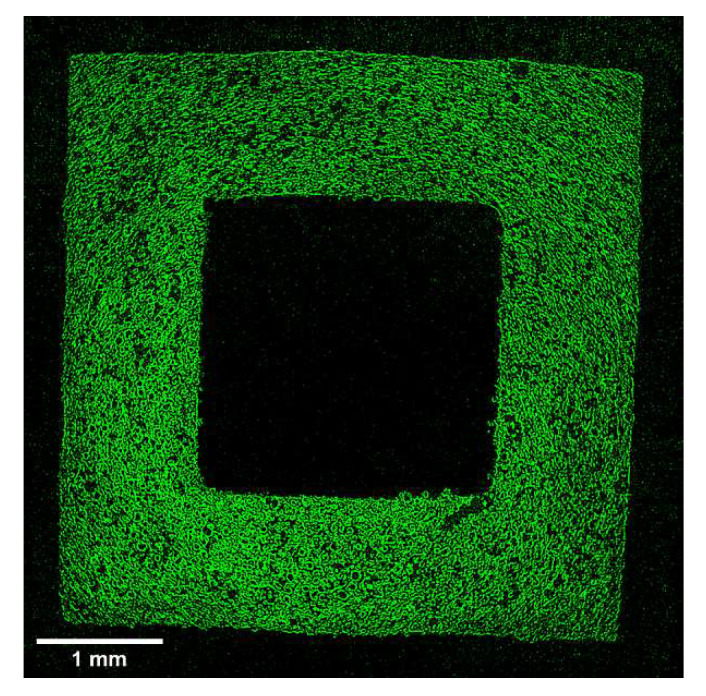
Cross-sectional X-ray tomography image of a 3D-printed hollow cubic carbon structure.

The average properties of the carbons prepared from the different resin formulations are presented in Table 3. The results show that slight differences in the acrylate ratio led to significant differences in the characteristics of the final carbon materials. The bulk density of the latter indeed ranged from 0.38 to 0.47 g·cm^−3^. All structures presented a high porosity, ranging from 0.72 to 0.79, with a similar pore size distribution (see Appendix A and Figure A1 and Figure A2), which significantly impacted the mechanical properties (see Table 3).

**Figure 5:**
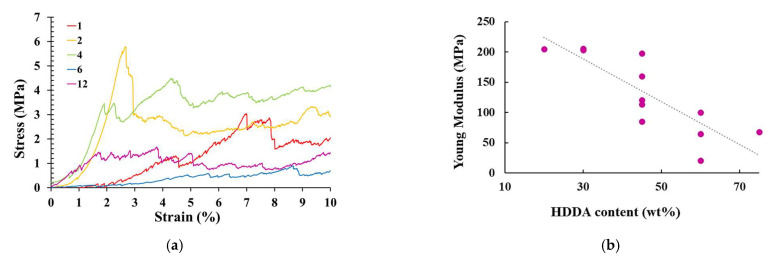
(**a**) Stress–strain curves of some 3D-printed carbon studied in this work. (**b**) Young modulus as a function of the initial HDDA content.

The shape of the compression curves shown in Figure 5a, where representative examples of some acrylate–tannin-derived carbons can be seen, suggesting, as expected, a typical elastic-brittle behaviour at relatively low strain, between 1.6% and 6%. After a linear part corresponding to the elastic deformation, the stress decreased sharply, thus, giving the compressive strength. This gave rise to a serrated stress–strain plateau, known as brittle crushing, followed by a progressive densification of the material at a strain of over 20% (not shown here).

Table 3 shows that the mechanical properties of the 3D-printed carbons produced in this work were higher than those of other carbons fabricated by stereolithography from purely synthetic precursors, which presented a calculated compressive strength of about 0.1 MPa [12]. Furthermore, the wide range of mechanical properties of these 3D-printed carbons obtained in a narrow density range is attractive compared to cellular monoliths, such as carbon foams with similar densities and those produced from tannin. Indeed, the latter monoliths have a narrower range of mechanical properties with, for example, a compressive strength ranging from 1 to 2 MPa for a broad variety of achievable densities between 0.1 and 0.25 g·cm^3^ [31,32]. 

In the broader context of bio-based porous carbon, our 3D-printed carbon showed a similar range of mechanical properties to sucrose-based monoliths made by compression moulding, with a compressive strength between 1.4 and 8.4 MPa depending on the amount of porogen and compression moulding parameters [33,34]. Likewise, the properties of our carbons are close to those of lignin-based carbon foams with a compressive strength ranging from 0.4 to 5 MPa, depending on the precursor and the foaming technique, for a total porosity between 70% and 80% [35,36,37]. 

These are rather good results, considering that sucrose and lignin-based monoliths have a carbon yield of about 40% and 50%, respectively, whereas our materials necessarily have a much lower yield due to the presence of acrylates, which are essential for photopolymerisation. This supports the multiple potentials of these tannin-based printed carbons, obtained in only two steps, and their use to develop materials under more or less demanding conditions, such as those in which the materials must be used in monolithic rather than powder form. Examples include electromagnetic wave absorbers and catalyst supports.

As expected, the mechanical properties depend on the resin composition, as we can observe a general reduction of the compressive strength and Young’s modulus with increasing HDDA content, see Table 3 and Figure 5b, while the different carbons show the same characteristics in terms of the pore size distribution. Indeed, carbons based on formulations containing 20–30 wt.% HDDA showed a Young’s modulus around 205 MPa, while the addition of the diluent in the resins led to structures with a much lower Young’s modulus fluctuating between 100 and 60 MPa for the formulations containing 45 wt.% and 60 wt.% HDDA, respectively. Thus, the HDDA content in the formulation has a significant influence on the mechanical properties of the pyrolysed structures. As a highly reactive acrylate diluent, HDDA is not known to be an excellent hardness promoter [38,39], which is supported by our observations.

At a given HDDA content, the correlation between higher density and higher compressive strength and moduli appears, which is characteristic of porous materials [40,41]. In addition, the printed architectures with the highest carbon yield were also those with the highest mechanical properties, while the dimensions of the pyrolysed structures were similar for the different formulations. Considering that the samples may present some point and surface defects after pyrolysis, the characterisation results obtained can be considered very reproducible.

### 3.2. Results of the Experimental Design and Related Statistical Analysis

The experimental design was exploited to model the correlations between the acrylate ingredients and the resultant properties of the 3D-printed porous carbons. The responses in terms of the carbon yield, compressive strength, and elastic modulus of the porous carbons were modelled as a function of the weight fractions of CN154CG, PETA, and HDDA in the resin formulation. A special Scheffé cubic mixture model (see Appendix B) was proposed by Design-Expert software according to Equations (4)–(6).

(4) $$\begin{array}{cc} \mathrm{Carbon}\;\mathrm{Yield} & =34.915\;A+46.456\;B+16.882\;C-91.991\;AB-19.410\;AC\\ & -43.285\;BC+148.027\;ABC \end{array}$$

(5) $$\begin{array}{cc} \mathrm{Compressive}\;\mathrm{Strength} & \\ & =27.092\;A+35.3998\;B+2.72289\;C-131.56\;AB\\ & -40.0722\;AC-58.2379\;BC+246.935\;ABC \end{array}$$

(6) $$\begin{array}{cc} \mathrm{Young}\;\mathrm{Modulus} & =673.687\;A+1037.56\;B+66.471\;C-3038.51\;AB\\ & -542.404\;AC-1505.21\;BC+4910.16\;ABC \end{array}$$

The statistical evaluation of the proposed special cubic model for fitting the carbon yield and mechanical properties was performed by analyses of variance (ANOVA). The quality of the fits was expressed by the coefficient of determination (R^2^), and the statistical significance of each parameter of the model was confirmed by Fisher’s F-tests [21,42]. More details on the statistics are given in Appendix B. Table 4 shows the ANOVA results for Equations (4)–(6).

The F-values 4.79, 5.76, and 15.64 gathered in Table 4 for the carbon yield, compressive strength, and Young’s modulus, respectively, are higher than the F-critical value (4.28) with a 95% confidence interval. This implies that the model applied is significant. Values of “Prob > F” < 0.050 also indicate that the model terms are significant. Thus, the coefficients AB and AC are never significant for the mechanical properties.

At this 95% confidence interval, the model terms are never significant for the carbon yield. However, reducing the model to a linear mixture model for this response does not improve the fit to the experimental data and the R^2^ drops. Thus, with a reduced confidence interval of 90%, the coefficients AB and AC are also not significant for the carbon yield response. Furthermore, as high R^2^ values correspond to good fits, values close to 1 illustrate the reliability of Equations (4)–(6). Herein, the R^2^ values were found to be 0.8274, 0.8521, and 0.9399 for the carbon yield, compressive strength, and Young’s modulus, respectively.

**Figure 6:**
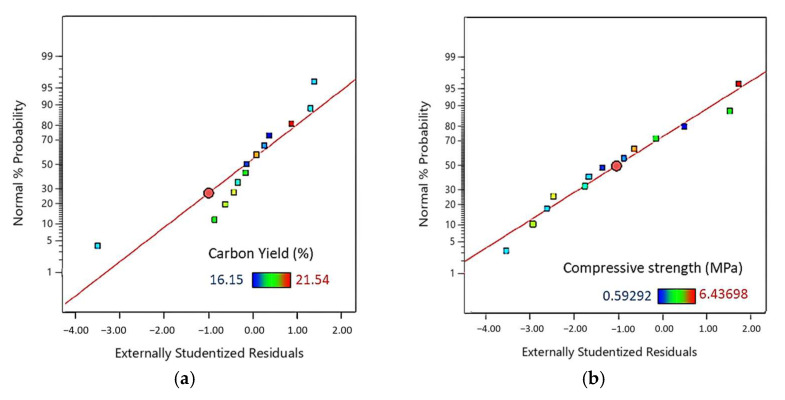

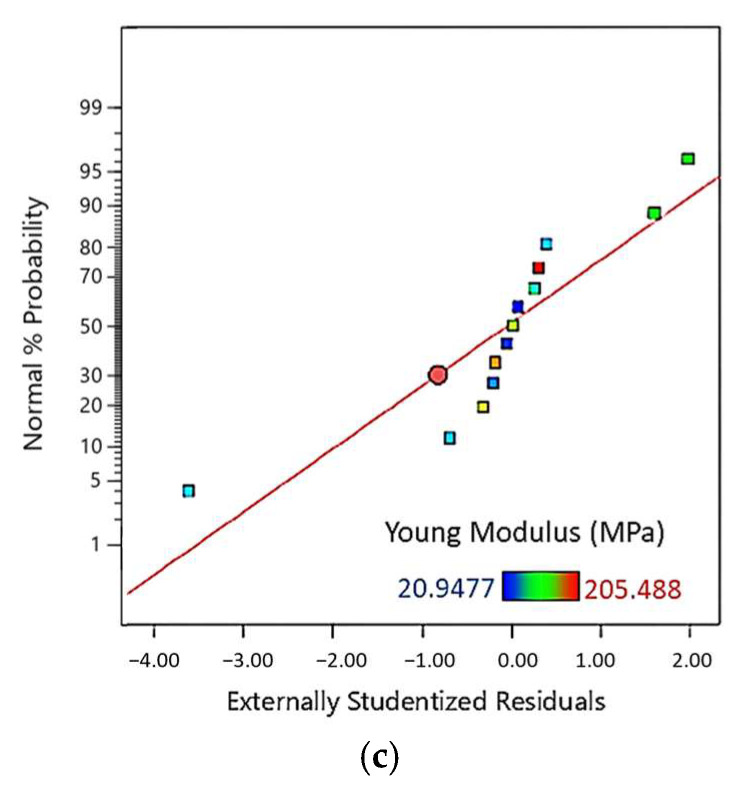
Normal probability plot for residuals for: the (**a**) carbon yield, (**b**) compressive strength, and (**c**) Young’s modulus. The numerical values for these properties increase from dark blue to red.

The normal plots of the residuals for the responses are presented in Figure 6, and the fact that most of the points are found on straight lines for the carbon yield and compressive strength indicates a reliable agreement between the experimental and calculated results. The relationship between the normal probability and studentized residuals for the Young’s modulus is approximately linear except for one outlier (formulation #11, with the most significant residuals compared to the model). Thus, after removing the outlier from the data set, we proceeded under the assumption that the error terms are normally distributed and that the experimental data and the calculated results are in good agreement.

### 3.3. Response Surface Methodology

**Figure 7:**
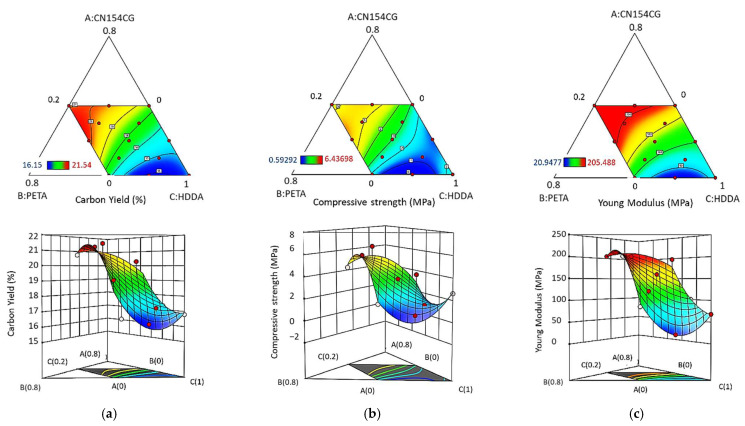
Contour plots (**top**) and surface plots (**bottom**) of the effect of the acrylate ratio on: the (**a**) carbon yield, (**b**) compressive strength, and (**c**) Young’s modulus. The numerical values of the responses increase from dark blue to red.

The relationship between the formulation parameters and responses is illustrated by contour plots and as three-dimensional response surface plots generated by the software. The influences of the three ingredients on the properties of the printed carbons are illustrated in Figure 7a–c. As shown in Figure 7b,c, the mechanical properties decreased drastically with the proportion of HDDA in the formulation, confirming the statement in Section 3.1, where we observed that the HDDA content drove the textural and mechanical properties.

Thus, we demonstrated here that the acrylate formulation in photocurable acrylate–tannin resins designed for the 3D printing of porous carbons strongly influences the mechanical properties of the pyrolysed structures. Hence, depending on the intended application and the mechanical stresses that a structure may be subjected to, a formulation can easily be determined by applying the model. In general, the interest is in highly resistant porous carbons that can be used under severe conditions. In order to illustrate the optimisation for this resin formulation, it was relevant to find an optimal combination of these acrylate ingredients with 25 wt.% of tannin offering the highest mechanical properties.

### 3.4. Optimisation of the Formulation

The resulting special cubic model, which determines the mechanical properties and carbon yield as a function of the acrylate ingredient ratio, was then applied to a desirability function. The latter is essentially a maximisation of all properties, i.e., a compressive strength higher than 5.5 MPa, a Young’s modulus higher than 205 MPa, and a carbon yield above 21.5%. These values correspond to formulations with a relatively low proportion of HDDA. 

In particular, two resin formulations with desirability above 90%—here called OF-1 (A: 0.27/B: 0.4/C: 0.33) and OF-2 (A: 0.4/B: 0.27/C: 0.33)—are expected to present such improved properties. Three specimens of each formulation were then examined, and their characteristics were measured and averaged. The corresponding results are shown in Table 5, together with the values predicted by the model. As can be seen, there is a satisfactory agreement between the measured and predicted values, thus, indicating the effectiveness of the model.

It is also interesting to note that not only do these carbons have a higher Young’s modulus compared with any of the tests reported in Table 3, they were also obtained in higher yields within the experimental uncertainties compared with any of the values in Table 3. Therefore, there is a real added value in having pushed the analysis to make such an optimisation, as it is a win–win situation.

## 4. Conclusions

Porous carbon architectures were obtained by stereolithography and the pyrolysis of acrylate–tannin resins. The properties of the resultant carbons (the yield, density, and mechanical properties) depended on their precursors. Therefore, it is interesting to understand the effect of the acrylate composition of the resin to optimise the formulation for a dedicated application. This study was conducted using an efficient tool: design of experiment (DOE) and related statistical analysis of the results (ANOVA). 

The results of the carbon yield and mechanical properties (i.e., the compressive strength and Young’s modulus) were modelled using a special Scheffé cubic mixture model. The latter allows the prediction of other materials’ properties within the investigated range of formulations. Different combinations of acrylate components with ratio constraints related to the processing conditions and fixed tannin content in the formulation were used to obtain 3D-printed carbon architectures with a wide range of mechanical properties. These formulations resulted in porous carbon structures with a yield of about 18%, a compressive strength between 0.5 and 5.7 MPa, and a Young’s modulus between 20 and 205 MPa. 

These properties were negatively affected by the increase in the hexanediol diacrylate (HDDA, the reactive acrylate diluent) content but remained close or even better than those of other closely related bio-based porous carbons of similar density.

The effectiveness of the DOE, and its related model, was tested by preparing printed porous carbons with the highest expected mechanical properties. The results showed that the corresponding properties were in excellent agreement with the predicted values and, above all, were better on average than the results used to create the experimental design. Thus, the model proved to be a valuable tool for developing 3D-printed porous carbons with customisable mechanical properties derived from acrylate–tannin resins.

## Figures and Tables

**Table 1:** Representative example of acrylate-resin formulation for 3D printing by SLA.

Component	Function	Content (wt.%)
Tannin	Bio-based carbon precursor	25
BAPO	Photoinitiator	0.3
CN154CG	Acrylated aromatic oligomer	29.9
PETA	Acrylated monomer	29.9
HDDA	Reactive diluent	14.9

**Table 2:** Our uniform experimental design scheme for the preparation of resins.

Formulation Number	Acrylate Component and Its Weight Fraction		
	CN154CG 0≤A≤0.4	PETA 0≤B≤0.4	HDDA 0.2 ≤C≤1
1	0	0	1
2	0.4	0	0.6
3	0	0.4	0.6
4	0.4	0.4	0.2
5	0.2	0	0.8
6	0	0.2	0.8
7	0.4	0.2	0.4
8	0.2	0.4	0.4
9	0.2	0.2	0.6
10	0.1	0.1	0.8
11	0.3	0.1	0.6
12	0.1	0.3	0.6
13	0.3	0.3	0.4

**Table 3:** The experimental results for the 13 printed carbons based on resins formulated after the mixture design.

No.	Carbon Yield (%)	*ρ_b_* (g·cm^−3^)	*ρ_s_* (g·cm^−3^)	*Φ* (-)	Compressive Strength (MPa)	Strain at Break (%)	Young’s Modulus (MPa)
1	16.8	0.411	1.620	0.746	2.6	3.72	67.9
2	18.9	0.467	1.624	0.732	3.6	3.92	197.7
3	19.1	0.399	1.694	0.764	1.6	2.55	85.3
4	20.8	0.404	1.847	0.781	4.3	1.67	205.0
5	17.3	0.447	1.656	0.730	1.1	2.24	100.2
6	16.2	0.385	1.779	0.784	0.6	5.90	20.9
7	20.5	0.422	1.755	0.759	4.7	3.88	205.5
8	21.3	0.432	1.756	0.757	5.7	4.62	203.3
9	18.6	0.433	1.720	0.748	3.5	1.66	160.0
10	17.2	0.427	1.793	0.767	1.3	6.01	65.0
11	20.3	0.457	1.716	0.734	1.8	3.58	113.6
12	16.4	0.463	1.666	0.722	1.7	1.96	120.5
13	21.5	0.419	1.689	0.752	6.4	4.94	203.9

**Table 4:** ANOVA results for Equations (4)–(6).

Source	Sum of Squares	Degree of Freedom	Mean Square	F-Value	Prob > F
ANOVA Carbon Yield					
Model	36.61	6	6.10	4.79	0.0390 *
^(1)^ Linear Mixture	31.51	2	15.75	12.38	0.0074 *
*AB*	2.76	1	2.76	2.17	0.1916
*AC*	0.7971	1	0.7971	0.6264	0.4588
*BC*	3.96	1	3.96	3.12	0.1280 ^x^
*ABC*	4.16	1	4.16	3.27	0.1207 ^x^
Residual	7.63	6	1.27		
Corrected Total Sum of Squares	44.24	12			
R-squared	0.8274				
ANOVA Compressive Strength					
Model	35.90	6	5.98	5.76	0.0256 *
^(1)^ Linear Mixture	22.04	2	11.02	10.61	0.0107 *
*AB*	4.78	1	4.78	4.60	0.0755
*AC*	3.40	1	3.40	3.27	0.1204
*BC*	7.18	1	7.18	6.91	0.0391 *
*ABC*	11.57	1	11.57	11.14	0.0157 *
Residual	6.23	6	1.04		
Corrected Total Sum of Squares	42.13	12			
R-Squared	0.8521				
ANOVA Young Modulus					
Model	47,545.51	6	7924.25	15.64	0.0020 *
^(1)^ Linear Mixture	41,471.29	2	20,735.65	40.93	0.0003 *
*AB*	2994.04	1	2994.04	5.91	0.0511
*AC*	622.50	1	622.50	1.23	0.3101
*BC*	4793.88	1	4793.88	9.46	0.0218 *
*ABC*	4573.96	1	4573.96	9.03	0.0239 *
Residual	3039.61	6	506.60		
Corrected Total Sum of Squares	50,585.12	12			
R-Squared	0.9399				

^(1)^*Y* = *αA* + *βB* + *γC*; * The coefficient is significant with a 95% confidence interval. ^x^ The coefficient is significant with a 90% confidence interval.

**Table 5:** Validation of the model: the measured values versus predicted values.

			Experimental				Predicted by the Model		
Sample	Desirability	*ρ_b_* (g·cm^−3^)	Carbon Yield (%)	Compressive Strength (MPa)	Young’s Modulus (MPa)		Carbon Yield (%)	Compressive Strength (MPa)	Young’s Modulus (MPa)
OF-1	0.952	0.465	23.09 ± 1.0	5.2 ± 0.3	216.7 ± 2.2		21.46	5.7	218.4
OF-2	0.905	0.450	22.68 ± 1.2	5.4 ± 0.1	211.4 ± 2.3	20	20.99	5.4	212.7

## Data Availability

Data is contained within the article.

## Sample Availability

Samples of 3D-printed resins and derived carbons are available from the authors.

## Appendix A

### Appendix A.1. Mercury Intrusion

Differential mercury intrusion curves were plotted as a function of the pore diameter. All pore size distributions were broad and multimodal. Modification of the acrylate formulation was not found to change the pore size distribution nor the median pore diameter with a value of about 32 µm (measured on material fragments).

### Appendix A.2. X-ray Tomography

Figure A2 shows a typical cross-sectional X-ray tomography image of a 3D-printed hollow cubic carbon structure. The pores, with an average diameter close to 35 µm, were well dispersed in the carbon structure. However, no significant differences were observed from sample to sample. These observations and measurements are in complete agreement with mercury intrusion.

## Appendix B

### Appendix B.1. Scheffé Mixture Models

In statistics, the response surface methodology explores the relationship between explanatory variables (*x_i_*, with *i* ranging from 1 to *q*, the number of variables) and one or more response variables (Ŷ). This relationship is described with a statistical model in the form of mathematical equations and can describe how a response variable Ŷ is influenced by the independent variables *x*_i_.

The simplest forms of statistical model introduced by Scheffé are polynomial models, such as the linear and special cubic models introduced in the present work:

Linear Model: $Ŷ=\overset{q}{\underset{i=1}{\sum }}\beta_{i}x_{i}$

Special Cubic Model: $Ŷ=\overset{q}{\underset{i=1}{\sum }}\beta_{i}x_{i}+\overset{q-1}{\underset{i<j}{\sum }}\overset{q}{\underset{j}{\sum }}\beta_{ij}x_{i}x_{j}+\overset{q-2}{\underset{i}{\sum }}\overset{q-1}{\underset{j<k}{\sum }}\overset{q}{\underset{k}{\sum }}\beta_{ijk}x_{i}x_{j}x_{k}$.

### Appendix B.2. Analysis of Variance (ANOVA)

Analysis of variance (ANOVA) is a set of analytical methods used to analyse the differences between means of values. In the mixture design, it is mainly used to test the effects of multiple factors (*x*, *y*, and *z*) and their interactions (*xy*, *xz*, *yz*, and *xyz*) on a measured quantity.

The Fisher F-test is recommended for comparing statistical models that have been fitted to a data set in order to identify the model that best fits that data population. The first step is to obtain the F-value from the data, which is defined as the ratio of the source mean square (also called variance) and the residual mean square:

$$F=s_{1}^{2}/s_{residuals}^{2}$$

The mean square is defined as $s_{i}^{2}=\sum^{\;}{\left(x_{i}-\bar{x_{i}}\right)}^{2}/\left(n-1\right)$ where (*n* − 1) is the degree of freedom. Then, to confirm or reject the null hypothesis (in our case, H0: “There is no factor effect”). Two methods can be applied.

The table method compares the calculated (or observed) value of F with the critical value determined from the Fisher table at a given significance level (*α*) and a given degree of freedom. If F > F_critical_, the null hypothesis is rejected, and the model is said to be significant.

When using the software method, the probability (Prob > F or *p*-value) of obtaining a value of F greater than or equal to the observed value is calculated. The null hypothesis is rejected if this probability is less than or equal to the significance level (*α*).
